# An energy harvester for all seasons

**DOI:** 10.1093/nsr/nwad218

**Published:** 2023-08-11

**Authors:** Qingping Wang, Chris Bowen

**Affiliations:** Department of Physics & Mechanical and Electronic Engineering, Hubei University of Education, China; Centre for Integrated Materials, Processes and Structures, Department of Mechanical Engineering, University of Bath, UK; Centre for Integrated Materials, Processes and Structures, Department of Mechanical Engineering, University of Bath, UK

**Figure 1.**
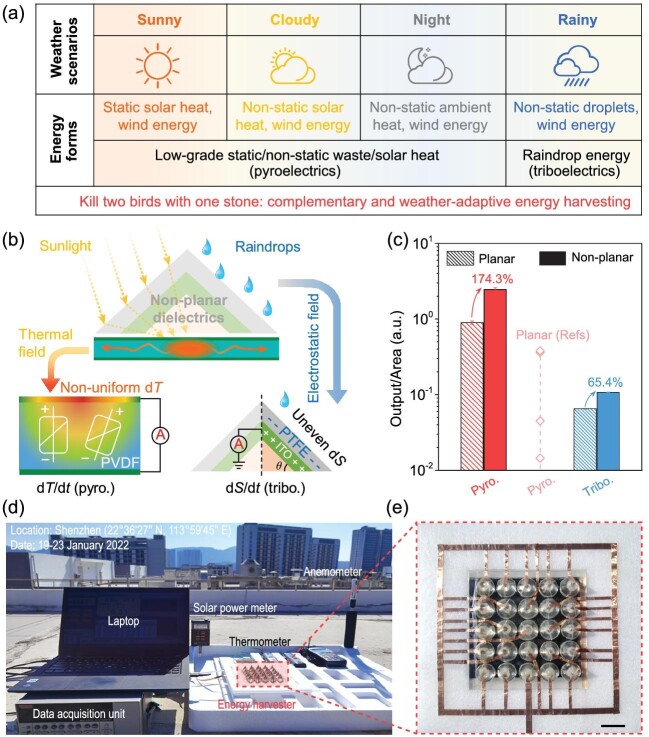
All-weather non-planar energy harvester. (a) Potential weather scenarios, where heat and wind induce pyroelectric effects, while rain droplets induce triboelectric effects. (b) Mechanism for a non-planar dielectric to produce rapid non-uniform temperature fluctuations (*dT/dt*, left) and rapid changes in droplet-solid contact area (*dS/dt*, right). (c) Comparison of output power for both planar and non-planar approaches. (d) Deployment of scaleable harvester, and corresponding (e) image of non-planar energy harvester; Scale bar: 2 cm [4].

The harvesting of sunlight, wind, heat and even raindrops to produce electrical energy has attracted significant attention to address the global energy crisis, reduce environmental pollution from fossil fuels, and limit the use of batteries [1–3]. One challenge to harvesting renewable energy sources is their inherent variability, where changes in weather can occur on a daily basis or as a result of diurnal cycles and seasonal changes (Fig. 1a).

The recent research by Zhou *et al.* [4]. has aimed to address this issue by creating an all-weather energy harvester based on a non-planar dielectric architecture to induce controlled changes in thermal and electrostatic fields (Fig. 1b, upper image). First, the high transmittance of the non-planar structure is able to focus sunlight to induce rapid in-plane temperature fluctuations within a pyroelectric element. The change in polarization of the pyroelectric leads to the generation of a charge, where the current is proportional to the rate of temperature change (d*T/*d*t*) (Fig. 1b, lower left). Second, the curved and textured nature of the non-planar dielectric leads to the spreading and separation of raindrops to produce rapid changes in droplet-solid contact area (d*S/*d*t*); see lower right Fig. 1b. The rapid change in droplet area acts to enhance the triboelectric output as a result of liquid-solid contact electrification. By taking this hybrid approach, this harvester was able to produce an enhanced electrical output due to a combination of a solar-induced pyroelectric response and raindrop-induced triboelectric effects.

As observed in Fig. 1c [4], their experimental results showed that the non-planar–based energy harvester enhanced pyroelectric output power by 174% at 0.2 sun, which surpassed conventional planar pyroelectrics that produce a more uniform thermal field. In addition, power generation from impacting rain droplets was increased by 65% compared to planar devices, which were less effective in facilitating droplet spreading and separation events.

The non-planar dielectric was designed to optimize the pyroelectric and triboelectric outputs by carefully tailoring its potential for sunlight transmission, sunlight concentration, and increasing droplet dynamics. The final structure was based on a transparent conical layered structure, which included polydimethylsiloxane (PDMS) as a conical lens to focus sunlight onto the pyroelectric element, a thin indium tin oxide (ITO) layer to act as a transparent conductor to collect the triboelectric charge, and a thin outer dielectric layer of hydrophobic polytetrafluoretyhylene (PTFE) that makes contact with the raindrops. The geometry of the conical lens was optimized to maximize the temperature fluctuations (d*T/*d*t*) in the pyroelectric, while the outer hydrophobic PTFE surface was optimized to improve droplet spreading (d*S/*d*t*) and charge transfer between the water droplet and the dielectric, without compromising the transmission of sunlight. The microscopic surface morphology and roughness of the PTFE also helped to enhance the triboelectric contribution. The pyroelectric energy harvesting element was based on a ferroelectric polyvinylidene fluoride (PVDF) film which was coated with carbon nanotubes (CNT) that acted as both an electrode and a solar absorber with desirable solar-to-heat conversion efficiency.

Outdoor testing of the non-planar dielectric for adaptive weather harvesting was undertaken, where the system is illustrated in Fig. 1d and e. In an outdoor setting, the temperature fluctuations were driven by both confined solar illumination and wind-/humidity-driven heat convection. Ambient convective heat variations also allow for power generation during night-conditions where solar cells are clearly unable to operate.

This innovative research provides a novel approach to all-weather energy harvesting, where the use of additive manufacturing provides a route for scalability. Potential applications can be the supply of power to sensor networks and the Internet of Things (IoT) [5].
